# *N*-Methyl-*N*-nitroso-*p*-toluene­sulfon­amide

**DOI:** 10.1107/S1600536814013518

**Published:** 2014-06-14

**Authors:** Kartik Rai, Vincent Wu, Priya Gupta, David A. Laviska, Benny C. Chan

**Affiliations:** a Department of Chemistry, The College of New Jersey, 2000 Pennington Rd, Ewing, NJ 08628, USA

## Abstract

The crystal structure of the title compound, C_8_H_10_N_2_O_3_S, displays predominant C—H⋯O hydrogen-bonding and π–π stacking inter­actions. The hydrogen bonds are between the O atoms of the sulfonyl group and H atoms on methyl groups. The π–π stacking inter­actions occur between adjacent aromatic rings, with a centroid–centroid distance of 3.868 (11) Å. These inter­actions lead to the formation of chains parallel to (101).

## Related literature   

For the use of the title compound as a nitro­sylating agent, see: Mayer *et al.* (2014 ▶). For related structures, see: Hakkinen *et al.* (1988 ▶); Lightfoot *et al.* (1993 ▶). For the use of the title compound as a potential cancer chemotherapeutic, see: Garcia-Rio *et al.* (2011 ▶); Skinner *et al.* (1960 ▶). For its use as an anti­microbial, see: Uri & Scola (1992 ▶) and as a precursor in methyl­ene production and production of heterocyclic rings, see: Hudlicky (1980 ▶). For literature hydrogen-bond lengths between sulfonyl O atoms and methyl H atoms in sulfonamide structures, see: Dodoff *et al.* (2004 ▶). For the potential use of sulfonamide compounds as ligands for metal coordination, see: Jacobs *et al.* (2013 ▶).
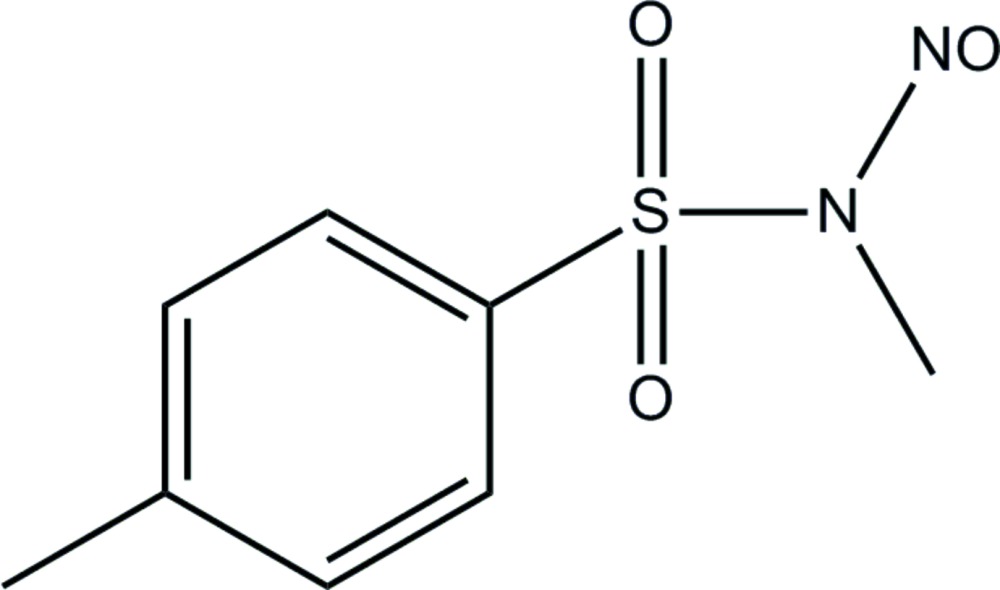


## Experimental   

### 

#### Crystal data  

C_8_H_10_N_2_O_3_S
*M* *_r_* = 214.24
Triclinic, 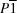

*a* = 6.8911 (8) Å
*b* = 8.4435 (10) Å
*c* = 8.6248 (10) Å
α = 81.458 (1)°
β = 85.883 (1)°
γ = 80.310 (1)°
*V* = 488.62 (10) Å^3^
*Z* = 2
Mo *K*α radiation
μ = 0.31 mm^−1^
*T* = 100 K
0.84 × 0.29 × 0.10 mm

#### Data collection  

Bruker APEXII CCD diffractometer
Absorption correction: numerical (*SADABS*; Bruker, 2011 ▶) *T*_min_ = 0.687, *T*_max_ = 0.746
5753 measured reflections
2275 independent reflections
1892 reflections with *I* > 2σ(*I*)
*R*_int_ = 0.024

#### Refinement  

*R*[*F* ^2^ > 2σ(*F* ^2^)] = 0.037
*wR*(*F* ^2^) = 0.096
*S* = 1.09
2275 reflections
137 parameters
H atoms treated by a mixture of independent and constrained refinement
Δρ_max_ = 0.36 e Å^−3^
Δρ_min_ = −0.37 e Å^−3^

### 

Data collection: *APEX2* (Bruker, 2011 ▶); cell refinement: *SAINT* (Bruker, 2011 ▶); data reduction: *SAINT*; program(s) used to solve structure: *SHELXS97* (Sheldrick, 2008 ▶); program(s) used to refine structure: *SHELXL2013* (Sheldrick, 2008 ▶); molecular graphics: *CrystalMaker* (CrystalMaker, 2009 ▶); software used to prepare material for publication: *publCIF* (Westrip, 2010 ▶).

## Supplementary Material

Crystal structure: contains datablock(s) global, I. DOI: 10.1107/S1600536814013518/fj2674sup1.cif

Structure factors: contains datablock(s) I. DOI: 10.1107/S1600536814013518/fj2674Isup2.hkl

Supporting information file. DOI: 10.1107/S1600536814013518/fj2674Isup3.cml

CCDC reference: 1007700

Additional supporting information:  crystallographic information; 3D view; checkCIF report

## Figures and Tables

**Table 1:** Hydrogen-bond geometry (Å, °)

*D*—H⋯*A*	*D*—H	H⋯*A*	*D*⋯*A*	*D*—H⋯*A*
C8—H8b⋯O1^i^	0.95 (2)	2.49 (2)	3.401 (2)	160

Symmetry code: (i) 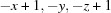.

**Fig. 1.**
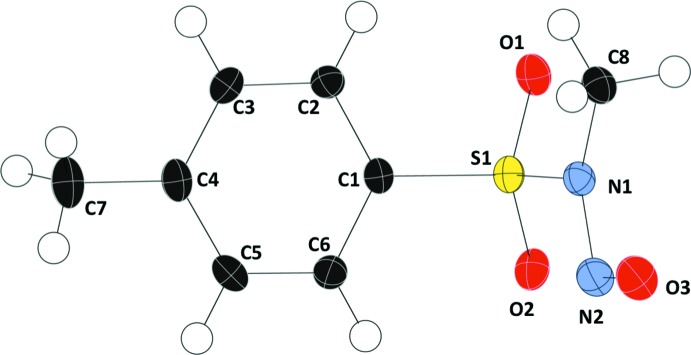
Thermal ellipsoid plot at 50% probability.

**Fig. 2.**
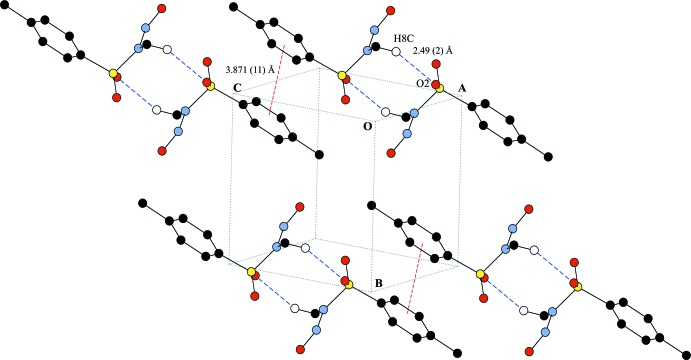
The title structure is stabilized by a hydrogen bond between O2 and H8C, which measures 2.49 (2) Å and pi-stacking interactions between adjacent benzene rings, which measures 3.871 (11) Å. Oxygen atoms are shown in red, carbon atoms in black, hydrogen atoms in white, and nitrogen atoms in blue. Symmetry equivalent pi-stacking and hydrogen bonding are indicated by red and blue dashed lines, respectively.

## supplementary crystallographic information

### S1. Comment

Diazald (*N*-methyl-*N*-nitroso-*p*- toluenesulfonamide) has been known to be a versatile reagent used in the general synthesis of diazomethane, a useful compound that serves as a precursor for methylene production and is used in the production of heterocyclic rings. (Hudlicky, 1980) Recently, these *N*-nitroso compounds have gained attention due to their potential cancer chemotherapeutic abilities. (Skinner *et al.*, 1960); (Garcia-Rio *et al.*, 2011) Additionally, the title compound was also found to behave as an antimicrobial agent against yeasts, fungi, Gram-negative, and Gram-positive bacteria. (Uri & Scola, 1992) The title compound was also shown to behave as a nitrosylating reagent in the formation of a new diruthenium complex. (Mayer *et al.*, 2014) Specifically, our group has investigated the potential of these sulfonamide structures as ligands for metal coordination. (Jacobs *et al.*, 2013) Here we report on the crystal structure of this versatile compound. This compound forms hydrogen bonds of 2.49 (2) Å between the oxygen atom (O1) on the sulfonyl group of one molecule and the hydrogen atom (H10B) on the methyl group of another. These hydrogen bond lengths were confirmed to be in the normal range (2.31 (6) Å - 2.53 (12) Å) between sulfonyl O atoms and methyl H atoms on sulfonamide structures. (Dodoff *et al.*, 2004) Additionally, pi-stacking interactions exist between adjacent aromatic rings and measure 3.868 (11) Å. These pi-stacking and hydrogen bonding interactions produce a stabilized dimerized crystal structure resulting in parallel chains.

### S2. Experimental

Approximately 100 mg of the title compound were dissolved in 2 ml of 100% isopropyl alcohol solution after being heated to boiling conditions. The solution was allowed to evaporate slowly for three days at approximately 4 C until clear, colorless crystals were formed. A crystal was manually separated and analyzed for crystallographic data using a Bruker APEXII CCD single-crystal X-ray diffractometer.

### S3. Refinement

The structure was solved using direct methods (Bruker, 2011). Hydrogen 8 A, 8B, 8 C were found by electron difference maps and then allowed to vary in 3 dimensions. The isotropic parameter was held to -1.2.

### Crystal data

**Table:**

C_8_H_10_N_2_O_3_S	*Z* = 2
*M**_r_* = 214.24	*F*(000) = 224
Triclinic, *P*1	*D*_x_ = 1.456 Mg m^−^^3^
*a* = 6.8911 (8) Å	Mo *K*α radiation, λ = 0.71073 Å
*b* = 8.4435 (10) Å	Cell parameters from 3237 reflections
*c* = 8.6248 (10) Å	θ = 2.5–28.1°
α = 81.458 (1)°	µ = 0.31 mm^−^^1^
β = 85.883 (1)°	*T* = 100 K
γ = 80.310 (1)°	Block, colorless
*V* = 488.62 (10) Å^3^	0.84 × 0.29 × 0.10 mm

### Data collection

**Table:**

Bruker APEXII CCD diffractometer	2275 independent reflections
Radiation source: fine-focus sealed tube	1892 reflections with *I* > 2σ(*I*)
Graphite monochromator	*R*_int_ = 0.024
Detector resolution: 8.3333 pixels mm^-1^	θ_max_ = 28.4°, θ_min_ = 2.4°
ω and φ scans	*h* = −9→9
Absorption correction: numerical (*SADABS*; Bruker, 2011)	*k* = −10→11
*T*_min_ = 0.687, *T*_max_ = 0.746	*l* = −11→11
5753 measured reflections	

### Refinement

**Table:**

Refinement on *F*^2^	Primary atom site location: structure-invariant direct methods
Least-squares matrix: full	Secondary atom site location: difference Fourier map
*R*[*F*^2^ > 2σ(*F*^2^)] = 0.037	Hydrogen site location: mixed
*wR*(*F*^2^) = 0.096	H atoms treated by a mixture of independent and constrained refinement
*S* = 1.09	*w* = 1/[σ^2^(*F*_o_^2^) + (0.0425*P*)^2^ + 0.2042*P*] where *P* = (*F*_o_^2^ + 2*F*_c_^2^)/3
2275 reflections	(Δ/σ)_max_ < 0.001
137 parameters	Δρ_max_ = 0.36 e Å^−^^3^
0 restraints	Δρ_min_ = −0.37 e Å^−^^3^

### Special details

**Table:**

Geometry. All e.s.d.'s (except the e.s.d. in the dihedral angle between two l.s. planes) are estimated using the full covariance matrix. The cell e.s.d.'s are taken into account individually in the estimation of e.s.d.'s in distances, angles and torsion angles; correlations between e.s.d.'s in cell parameters are only used when they are defined by crystal symmetry. An approximate (isotropic) treatment of cell e.s.d.'s is used for estimating e.s.d.'s involving l.s. planes.
Refinement. Refinement of *F*^2^ against ALL reflections. The weighted *R*-factor *wR* and goodness of fit *S* are based on *F*^2^, conventional *R*-factors *R* are based on *F*, with *F* set to zero for negative *F*^2^. The threshold expression of *F*^2^ > σ(*F*^2^) is used only for calculating *R*-factors(gt) *etc*. and is not relevant to the choice of reflections for refinement. *R*-factors based on *F*^2^ are statistically about twice as large as those based on *F*, and *R*- factors based on ALL data will be even larger.

### Fractional atomic coordinates and isotropic or equivalent isotropic displacement parameters (Å^2^)

**Table:**

	*x*	*y*	*z*	*U*_iso_*/*U*_eq_	
S1	0.20728 (6)	0.03322 (5)	0.28268 (5)	0.01794 (13)	
O1	0.33666 (19)	−0.07228 (14)	0.38895 (14)	0.0234 (3)	
O2	0.05198 (18)	−0.02289 (15)	0.21663 (15)	0.0239 (3)	
O3	−0.1436 (2)	0.37946 (16)	0.40008 (15)	0.0301 (3)	
N1	0.0960 (2)	0.18010 (17)	0.38888 (16)	0.0184 (3)	
N2	−0.0760 (2)	0.26414 (19)	0.33146 (18)	0.0246 (3)	
C1	0.3438 (2)	0.13715 (19)	0.13431 (19)	0.0167 (3)	
C2	0.5359 (3)	0.1532 (2)	0.1589 (2)	0.0200 (4)	
H2	0.5971	0.1025	0.2528	0.024*	
C3	0.6369 (3)	0.2450 (2)	0.0434 (2)	0.0219 (4)	
H3	0.7691	0.2557	0.0584	0.026*	
C4	0.5484 (3)	0.3215 (2)	−0.0938 (2)	0.0204 (4)	
C5	0.3552 (3)	0.3032 (2)	−0.1148 (2)	0.0220 (4)	
H5	0.2932	0.3554	−0.2079	0.026*	
C6	0.2519 (3)	0.2107 (2)	−0.0028 (2)	0.0204 (4)	
H6	0.1209	0.1976	−0.0190	0.024*	
C7	0.6590 (3)	0.4220 (2)	−0.2174 (2)	0.0282 (4)	
H7A	0.7301	0.4901	−0.1669	0.042*	
H7B	0.5657	0.4911	−0.2891	0.042*	
H7C	0.7532	0.3502	−0.2764	0.042*	
C8	0.1981 (3)	0.2380 (2)	0.5075 (2)	0.0219 (4)	
H8A	0.194 (3)	0.352 (3)	0.480 (2)	0.033*	
H8B	0.329 (3)	0.181 (3)	0.512 (2)	0.033*	
H8C	0.137 (3)	0.215 (3)	0.609 (3)	0.033*	

### Atomic displacement parameters (Å^2^)

**Table:**

	*U*^11^	*U*^22^	*U*^33^	*U*^12^	*U*^13^	*U*^23^
S1	0.0207 (2)	0.0151 (2)	0.0183 (2)	−0.00448 (16)	0.00224 (16)	−0.00297 (15)
O1	0.0286 (7)	0.0165 (6)	0.0228 (6)	−0.0012 (5)	0.0013 (5)	0.0012 (5)
O2	0.0252 (7)	0.0247 (7)	0.0254 (7)	−0.0118 (5)	0.0032 (5)	−0.0084 (5)
O3	0.0338 (8)	0.0250 (7)	0.0277 (7)	0.0064 (6)	0.0014 (6)	−0.0053 (6)
N1	0.0195 (7)	0.0190 (7)	0.0165 (7)	−0.0018 (6)	0.0003 (6)	−0.0037 (6)
N2	0.0249 (8)	0.0241 (8)	0.0223 (8)	0.0016 (6)	−0.0004 (6)	−0.0019 (6)
C1	0.0203 (8)	0.0131 (8)	0.0170 (8)	−0.0030 (6)	0.0025 (6)	−0.0040 (6)
C2	0.0186 (8)	0.0207 (9)	0.0203 (8)	−0.0015 (7)	−0.0015 (7)	−0.0026 (7)
C3	0.0177 (9)	0.0226 (9)	0.0259 (9)	−0.0052 (7)	0.0016 (7)	−0.0039 (7)
C4	0.0274 (9)	0.0148 (8)	0.0192 (8)	−0.0039 (7)	0.0064 (7)	−0.0062 (7)
C5	0.0298 (10)	0.0199 (9)	0.0158 (8)	−0.0020 (7)	−0.0025 (7)	−0.0020 (7)
C6	0.0197 (9)	0.0226 (9)	0.0197 (9)	−0.0038 (7)	−0.0010 (7)	−0.0056 (7)
C7	0.0372 (11)	0.0228 (10)	0.0251 (10)	−0.0092 (8)	0.0092 (8)	−0.0046 (8)
C8	0.0269 (10)	0.0215 (9)	0.0187 (9)	−0.0063 (8)	−0.0008 (7)	−0.0046 (7)

### Geometric parameters (Å, º)

**Table:**

S1—O2	1.4258 (13)	C3—H3	0.9500
S1—O1	1.4268 (13)	C4—C5	1.393 (3)
S1—N1	1.6975 (14)	C4—C7	1.506 (2)
S1—C1	1.7504 (17)	C5—C6	1.384 (2)
O3—N2	1.2224 (19)	C5—H5	0.9500
N1—N2	1.360 (2)	C6—H6	0.9500
N1—C8	1.466 (2)	C7—H7A	0.9800
C1—C2	1.388 (2)	C7—H7B	0.9800
C1—C6	1.394 (2)	C7—H7C	0.9800
C2—C3	1.390 (2)	C8—H8A	0.96 (2)
C2—H2	0.9500	C8—H8B	0.95 (2)
C3—C4	1.391 (2)	C8—H8C	0.96 (2)
			
O2—S1—O1	121.43 (8)	C3—C4—C7	120.73 (17)
O2—S1—N1	105.80 (7)	C5—C4—C7	120.63 (16)
O1—S1—N1	104.15 (7)	C6—C5—C4	121.35 (16)
O2—S1—C1	109.93 (8)	C6—C5—H5	119.3
O1—S1—C1	110.06 (8)	C4—C5—H5	119.3
N1—S1—C1	103.75 (7)	C5—C6—C1	118.66 (16)
N2—N1—C8	121.69 (14)	C5—C6—H6	120.7
N2—N1—S1	114.33 (11)	C1—C6—H6	120.7
C8—N1—S1	122.36 (12)	C4—C7—H7A	109.5
O3—N2—N1	113.28 (14)	C4—C7—H7B	109.5
C2—C1—C6	121.41 (16)	H7A—C7—H7B	109.5
C2—C1—S1	119.72 (13)	C4—C7—H7C	109.5
C6—C1—S1	118.76 (13)	H7A—C7—H7C	109.5
C1—C2—C3	118.62 (16)	H7B—C7—H7C	109.5
C1—C2—H2	120.7	N1—C8—H8A	108.3 (13)
C3—C2—H2	120.7	N1—C8—H8B	108.9 (13)
C2—C3—C4	121.31 (16)	H8A—C8—H8B	112.2 (19)
C2—C3—H3	119.3	N1—C8—H8C	110.6 (13)
C4—C3—H3	119.3	H8A—C8—H8C	110.3 (18)
C3—C4—C5	118.64 (16)	H8B—C8—H8C	106.5 (18)
			
O2—S1—N1—N2	−30.84 (14)	O1—S1—C1—C6	162.52 (13)
O1—S1—N1—N2	−159.92 (12)	N1—S1—C1—C6	−86.55 (14)
C1—S1—N1—N2	84.88 (13)	C6—C1—C2—C3	0.0 (2)
O2—S1—N1—C8	163.44 (13)	S1—C1—C2—C3	−175.94 (13)
O1—S1—N1—C8	34.37 (15)	C1—C2—C3—C4	0.8 (3)
C1—S1—N1—C8	−80.84 (15)	C2—C3—C4—C5	−0.6 (3)
C8—N1—N2—O3	−7.3 (2)	C2—C3—C4—C7	179.38 (16)
S1—N1—N2—O3	−173.13 (12)	C3—C4—C5—C6	−0.3 (3)
O2—S1—C1—C2	−157.71 (13)	C7—C4—C5—C6	179.71 (16)
O1—S1—C1—C2	−21.41 (16)	C4—C5—C6—C1	1.0 (3)
N1—S1—C1—C2	89.53 (14)	C2—C1—C6—C5	−0.9 (3)
O2—S1—C1—C6	26.22 (15)	S1—C1—C6—C5	175.10 (13)

### Hydrogen-bond geometry (Å, º)

**Table:**

*D*—H···*A*	*D*—H	H···*A*	*D*···*A*	*D*—H···*A*
C8—H8b···O1^i^	0.95 (2)	2.49 (2)	3.401 (2)	160

Symmetry code: (i) −*x*+1, −*y*, −*z*+1.
